# White matter abnormalities and cognitive function in euthymic patients with bipolar disorder and major depressive disorder

**DOI:** 10.1002/brb3.1868

**Published:** 2020-10-03

**Authors:** Yoshikazu Masuda, Go Okada, Masahiro Takamura, Chiyo Shibasaki, Atsuo Yoshino, Satoshi Yokoyama, Naho Ichikawa, Shiho Okuhata, Tetsuo Kobayashi, Shigeto Yamawaki, Yasumasa Okamoto

**Affiliations:** ^1^ Department of Psychiatry and Neuroscience Hiroshima University Hiroshima Japan; ^2^ Graduate School of Engineering Kyoto University Kyoto Japan

**Keywords:** bipolar disorder, diffusion tensor imaging, euthymic state, major depressive disorder, sustained attention, white matter

## Abstract

**Objectives:**

In recent years, a growing number of diffusion tensor imaging (DTI) studies have compared white matter integrity between patients with major depressive disorder (MDD) and bipolar disorder (BD). However, few studies have examined the pathophysiological significance of different degrees of white matter abnormalities between the two disorders. The present study comprehensively assessed white matter integrity among healthy controls (HC) and euthymic patients with MDD and BD using whole‐brain tractography and examined associations between white matter integrity and cognitive functioning.

**Methods:**

We performed neurocognitive examinations and DTI with 30 HCs, 30 patients with MDD, and 30 patients with BD. We statistically evaluated white matter integrity and cognitive function differences across the three groups, assessing associations between white matter integrities and cognitive function.

**Results:**

The BD group showed lower fractional anisotropy (FA) for the corpus callosum body, as well as lower, sustained attention and set‐shifting scores compared to the other groups. FA for the left body of the corpus callosum was correlated with sustained attention in patients with BD.

**Conclusions:**

The significant reduction of white matter integrity in the corpus callosum in BD, compared to MDD, was associated with an impairment of sustained attention. This result promotes the understanding of the significance of white matter integrity in mood disorders.

## Graphical abstract

The BD shows greater reduction of white matter integrity in body of the corpus callosum compared to the MDD, and the abnormality correlates with dysfunction in sustained attention.

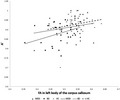

## 1 INTRODUCTION

Major depressive disorder (MDD) and bipolar disorder (BD) show multiple domains of cognitive impairment, including such domains as memory, attention, processing speed, and executive function (Marvel & Paradiso, 2004). Some impairments remain even during euthymic periods and are thought to affect the patient's psychosocial and occupational outcomes regardless of psychiatric symptoms (Bora et al., 2013; Lam et al., 2014; Martinez‐Aran et al., 2004; Robinson et al., 2006). Comparing effect sizes suggest that the degree of impairment is greater in patients with BD than those with MDD (MacQueen & Memedovich, 2017). Moreover, although some direct comparisons of impairments between patients with MDD and BD have reported no differences (Daniel et al., 2013; Hermens et al., 2010), other studies have reported greater impairment in patients with BD in domains such as attention and executive function (Benson et al., 2008; Borkowska & Rybakowski, 2001; Cai et al., 2012; Clark et al., 2005; Cotrena et al., 2016; Iverson et al., 2011; Maalouf et al., 2010; Poletti et al., 2017; Xu et al., 2012). Therefore, cognitive impairment profiles may be different between MDD and BD patients.

Recently, the neural basis of cognitive functioning has been clarified using neuroimaging techniques. In particular, studies on the associations between cognitive function and white matter connectivity using the diffusion tensor imaging (DTI) technique, which enables the evaluation of white matter microstructures by quantifying the extension and direction of diffusion in water molecules, (Le Bihan, (2006)) have received increased attention. A large number of studies have indicated associations between white matter microstructures and age‐related degenerations (Bennett & Madden, 2014; Madden et al., 2009) or training effects (Sampaio‐Baptista & Johansen‐Berg, 2017) on the cognitive functions of healthy individuals, and a broad range of white matter tracts are thought to be associated with different cognitive functions. Furthermore, many studies have reported that cognitive dysfunctions in patients with Alzheimer's disease (Acosta‐Cabronero & Nestor, 2014) or schizophrenia (Karlsgodt, 2016) correlate with reduced white matter integrity.

Patients with MDD and BD show abnormal white matter integrity across multiple white matter tracts including the cingulate bundle, corpus callosum, and the superior longitudinal fasciculus (Bracht et al., 2015a; Chen et al., 2016; Duarte et al., 2016; Kelly et al., 2017; Liao et al., 2013; Nortje et al., 2013; Pauling et al., 2017). These abnormalities are thought to be associated with cognitive impairments (Alves et al., 2012; Bauer et al., 2015; Dalby et al., 2012; Kafantaris et al., 2009; Knöchel et al., 2016, 2014; Li et al., 2014; Linke et al., 2013; Liu et al., 2010; Magioncalda et al., 2016; McKenna et al., 2015; Murphy et al., 2007; Oertel‐Knöchel et al., 2014; Poletti et al., 2015; Rizk et al., 2017; Yin et al., 2016). Direct comparison of white matter integrities between patients with MDD and BD has shown more abnormalities in patients with BD, in tracts such as the corpus callosum, cingulate bundle, uncinate fasciculus, thalamic radiation, superior longitudinal fasciculus, and inferior longitudinal fasciculus (Benedetti et al., 2011; Deng et al., 2018; Matsuoka et al., 2017; Repple et al., 2017; Versace et al., 2010). However, few studies have examined the pathophysiological significance of different degrees of white matter abnormalities between the two disorders.

Yamada et al. (2015) is the only study to date that directly compared possible relationships between white matter integrity and cognitive function in patients with MDD and BD. They found that both groups show cognitive dysfunctions in working memory and attention, along with white matter abnormalities in the anterior part of the corpus callosum, though only patients with MDD showed a correlation between white matter integrity and working memory/attention performance. However, Yamada et al. only examined the corpus callosum. Therefore, it remains unclear how other white matter tracts might be associated with cognitive functions in these disorders. In addition, the mood state was not controlled in their study, which could have some effects on white matter integrity and cognitive function. In other words, changes in white matter integrity and cognitive function could be both state markers reflecting the state or the severity of current symptoms and a trait marker reflecting the vulnerability to persistent mood abnormalities. Thus, for understanding mood disorders, it is essential to clarify whether abnormalities in cognition and white matter are caused by the current symptoms or persistent changes resulting from the disorders themselves (Dvorak et al., 2019).

Therefore, we examined 54 whole‐brain white matter tracts in patients with MDD, BD, and healthy controls (HCs), using an exploratory tractographic approach following the method described by Okuhata et al. (2018) and Okuhata et al. (2018). We also restricted the patients’ current mood state to euthymia. As mentioned above, studies focusing on euthymic individuals have demonstrated disease‐specific and persistent neural changes that reflect the vulnerability to mood abnormalities, which might ultimately help understand the pathophysiology of mood disorders. We hypothesized that euthymic patients with BD would show greater abnormalities in both white matter integrities and cognitive functions compared to euthymic patients with MDD, and that white matter abnormality would be related to cognitive dysfunctions.

## 2 METHOD

### 2.1 Participants

The study included 30 individuals with MDD, 30 individuals with BD (type I = 23 and type II = 7), and 30 age‐matched HCs. The participants had been previously diagnosed by an expert clinician using the DSM‐5. The Japanese version of Mini‐International Neuropsychiatric Interview (MINI) was performed at the time of participation to confirm the diagnosis (Otsubo et al., 2005). HCs were also screened with the MINI to confirm that they have no psychiatric disorder. All the participants were right‐handed native speakers of Japanese. Participants were excluded if they had (a) a comorbid diagnosis of schizophrenia, alcohol or substance abuse/dependence, dementia, development disorders, eating disorders, or personality disorders, (b) severe physical illness, (c) high‐level suicide risk, and (d) currently breastfeeding during pregnancy or the postpartum period.

We assessed the symptom severity of the patient groups using the Hamilton Rating Scale for Depression‐17 items (HAMD) and the Young Mania Rating Scale (YMRS). Moreover, the symptom severity of each participant on the day of the MRI scan was assessed by using self‐assessment scales, including the Beck Depression Inventory‐Ⅱ (BDI‐Ⅱ) and the Altman Self Rating Mania Scale (ASRM). In addition, we estimated the premorbid intelligence quotients of the participants by using the Japanese Adult Reading Test‐25 (JART). Euthymia was defined as a YMRS total score < 8 and a HAMD cutoff values < 8. All patients were euthymic at the time of the MRI scan according to this criterion.

### 2.2 Neurocognitive assessment

Neurocognitive assessments of the three groups were conducted by using the following four tasks in the Cambridge Neuropsychological Test Automated Battery (CANTAB, Cambridge Cognition, Cambridge, UK) (Sahakian & Owen, 1992).

Reaction time (RTI) is a test for evaluating psychomotor speed by measuring the time during which the subject responds to the target. In this test, the participant must hold down a button at the bottom of a screen. Five circles are presented on the screen. In each case, a yellow dot appears in one of the circles, and the participant must touch that yellow dot as soon as possible. Median five‐choice RTIs and median five‐choice movement times were analyzed. Median RTI is the median duration between the onset of the stimulus and the time at which the subject released the button. Median movement time is the median time taken to touch the stimulus after the button has been released.

Rapid visual processing (RVP) evaluates sustained attention by requiring participants to detect a series of target sequences of digits (e.g., 2,4,6; 4,6,8; 3,5,7) among pseudorandomly appearing digits at the rate of 100 digits per minute. Digits appear in a white box in the center of a screen, and participants must register their responses by touching a button. A′ and median response latency were analyzed. A′ (A prime) is a signal detection measure of the sensitivity to the target, regardless of the response tendency (the expected range is 0.00 to 1.00; bad to good). In essence, this metric is a measure of how good a participant is at detecting a target against ongoing distractions.

Intra/extradimensional shift task (IED) evaluates cognitive flexibility, analogous to the Wisconsin Card Sorting Test, by assessing rule learning and reversal. In this test, two patterns of figures are presented on the screen, which includes a white line and color‐filled shapes. Participants are instructed to select the correct figure based on an underlying rule, which is changed after six continuous correct responses. The rules involve two dimensions. In the intradimensional shift stage, participants keep responding to a relevant stimulus (e.g., the shape remains the relevant set, but a different shape is now correct). Moreover, in the extradimensional shift stage, participants need to shift their attention to an irrelevant stimulus (e.g., shapes are no longer the relevant set; instead, one of the line stimuli is now correct). The task consists of nine stages, such that the intradimensional shifts and reversals are included through the series of seven stages, and the final two stages include extradimensional shifts and reversals. The task is automatically terminated if the participants fail to select six continuous responses after 50 trials at any stage. Total errors adjusted and stages completed were analyzed. Total errors adjusted are the total number of errors made in the trials throughout the task. Stages completed are the successful completion of one of the set tasks.

Spatial working memory (SWM) is a self‐ordered search task for evaluating SWM. In this test, a number of colored boxes are presented on the screen. The participants are required to search for tokens hidden in one of the boxes. They are informed that the tokens are hidden one at a time and that tokens are never hidden in the same box twice over the course of one trial. Therefore, the participants must do multiple searches within one trial while avoiding previous token locations. The number of boxes is gradually increased up to eight. Between‐errors and the strategy used are analyzed in this test. Between‐errors are the times the participant revisits a box in which a token has previously been found. A strategy is the number of distinct boxes used by the participant to begin a new search for a token, within the same problem.

### 2.3 MRI data acquisition

MRI scans were performed at Hiroshima University in Hiroshima, Japan, using a 3.0T Siemens Magnetom (Siemens, Munich, Germany) with a 12‐channel head coil. Thirty noncollinear direction DTI data sets were acquired with the following sequence parameters: 60 slices, TR/TE = 8100/94 ms, FOV = 240 mm, voxel size = 2.5 × 2.5 × 2.5 mm^3^, image matrix = 96 × 96. The b value was *b* = 1,000 s/mm^2^, and one image was also acquired with *b* = 0.

### 2.4 Data preprocessing and fiber tracking

We conducted data preprocessing and atlas‐based whole‐brain fiber tracking based on the method described by Okuhata et al. (2018) We used the Functional MRI of the Brain (FMRIB) Software Library for data preprocessing. First, nonbrain tissue was deleted with the brain extraction tool from eddy current corrected diffusion MRI data. We then calculated diffusion indices such as FA, tensors, and the first eigenvector by using the FMRIB Diffusion Toolbox. Finally, we performed linear and nonlinear registrations using the FMRIB Linear Image Registration Tool, followed by the FMRIB Nonlinear Image Registration Tool. We performed the automated fiber tracking with the tensor deflection method for the 54 white matter parcels, which were prescribed using the Johns Hopkins University Diffusion Tensor Imaging (JHU DTI)‐based white matter atlas. We calculated the FA value at each stepping point (stepping width: 0.5 mm) along each fiber via interpolation, using the volume data for the center points of the nearest eight voxels around the step point. The criteria for terminating were as follows: FA < 0.25 and flip angle > 45°. Fiber tracking procedures were performed using MATLAB for Windows (ver. R2015b; The MathWorks, Inc.).

### 2.5 Statistical analysis

One‐way analysis of variance (ANOVA) was used to analyze the variables, including the estimated IQ, BDI‐Ⅱ, and ASRM scores. Moreover, a chi‐square test was used for comparing the gender distribution, as well as demographic and clinical characteristics of the participants across the three groups. Independent‐sample *t* tests were used for comparing the duration of illness, the number of depression episodes, the HAMD score, and the YMRS score, as well as the clinical characteristics of participants with MDD and BD. Analysis of covariate (ANCOVA) and Bonferroni's post hoc analyses were used to compare the outcomes of neurocognitive assessments across the three groups with age, gender, duration of illness, and the number of depression episodes as covariates. We used “0” for variables that were not accessible in the healthy group, including the illness duration, and episodes of depression. The significance level for the statistical analyses, including demographics, clinical characteristics, neurocognitive assessment outcomes, and post hoc analysis, was set as *p* < .05. Differences in the mean FA of 54 white matter tracts between the three groups were assessed using an ANCOVA with age, gender, duration of illness, and the number of depression episodes as covariates. Bonferroni's correction was used in the analyses to correct for multiple comparisons. The significant level was set as *p* < .001, which is approximate to 0.05/54 (*n* = 54). Bonferroni's post hoc tests were conducted with the significance level set at *p* < .05. Correlation analyses of neurocognitive assessment outcomes, as well as the mean FA of tracts in which significant differences were recognized, were conducted using Pearson's correlation test for all participants and separately each of the three groups. The significance level for these tests was set at *p* < .05. We used IBM SPSS Statistics version 24 for Windows (SPSS Japan Inc., Tokyo, Japan) for all statistical analyses.

### 2.6 Ethical approval

The Ethics Committee of the Medical Faculty of Hiroshima University approved this study, and it was conducted according to the Helsinki Declaration of 1975. Prior to participation in the study, participants gave their written informed consent and received financial compensation for their participation.

## 3 RESULTS

### 3.1 Demographics and clinical characteristics

Summaries of demographics and clinical characteristics are provided in Table 1. All patients were euthymic at the time of the MRI scan (HAMD < 8; YMRS < 8). There were no significant differences in age, gender distribution, estimated IQ, BDI‐Ⅱ score, and ASRM score across the three groups. Groups were matched on HAMD and YMRS scores. The participants with BD had significantly longer illness duration times (MDD = 8.6 years, BD = 19.7 years, t = 3.946, *p* < .001) and had a greater number of depressive episodes (MDD = 3.2, BD = 10.8, t = 7.540, *p* < .001) than the participants with MDD. All patients were on medication at the time of the MRI scan. Medication details are provided in Table 1.

**Table 1:** Demographics, clinical characteristics, and psychiatric medication for study participants

	MDD (*n* = 30)	BD (*n* = 30)	HC (*n* = 30)	Statistics	*p*
Age	54.0 ± 12.4	50.8 ± 14.9	52.2 ± 15.3	*F* = 0.371	.691
Gender (male/female, *n*)	11/19	15/15	13/17	*χ* ^2^ = 1.086	.581
BDI‐Ⅱ	9.5 ± 7.5	9.4 ± 7.8	7.5 ± 6.3	*F* = 0.735	.482
ASRM	1.5 ± 1.7	0.9 ± 0.9	1.6 ± 2.5	*F* = 1.166	.316
HRSD	3.0 ± 2.1	3.8 ± 3.1		t = 1.229	.224
YMRS	0.8 ± 1.3	1.4 ± 1.8		t = 1.592	.117
Duration of illness, yr	8.6 ± 9.8	19.7 ± 14.3		t = 3.946	<.001
Number of depressive episodes	3.2 ± 4.2	8.9 ± 7.8		t = 3.555	<0.001
Estimated IQ	105.7 ± 9.2	105.8 ± 11.0	106.1 ± 7.9	*F* = 0.016	.984
Lithium, % (*n*)	13.3 (4)	76.7 (23)			
Antidepressant, % (*n*)	93.3 (28)	33.4 (10)			
Anticonvulsant, % (*n*)	0 (0)	63.3 (19)			
Antipsychotic, % (*n*)	13.3 (4)	33.4 (10)			
Benzodiazepine, % (*n*)	70.0 (21)	40.0 (12)			

Abbreviations: ASRM, Altman Self Rating Mania Scale; BD, bipolar disorder; BDI‐Ⅱ, Beck Depression Inventory‐Ⅱ; HC, healthy control; HRSD, Hamilton Rating Scale for Depression; MDD, major depressive disorder; *n*, number; *SD*, standard deviation; YMRS, Young Mania Rating Scale; yr, year.

### 3.2 DIfference in neurocognitive functions between groups

There were significant main effects of the group for the raw five‐choice RTI scores and five‐choice movement time scores in RTI, A′ in RVP, total errors adjusted in IED, and strategy in SWM across the three groups. Post hoc group comparisons indicated that five‐choice RTIs were significantly longer in the participants with MDD and BD comparing to the HCs (MDD > HC: *p* = .027; BD > HC: *p* = .016), and A′ in RVP (MDD > BD: *p* = .041; HC > BD: *p* = .009) and total errors adjusted in IED (MDD > BD: *p* = .006; HC > BD: *p* = .002) were worse in the participants with BD compared to the participants with MDD and HCs. No significant difference was found in five‐choice movement time scores in RTI (MDD vs. HC: *p* = .167; BD vs. HC: *p* = .711; MDD vs. BD: *p* = 1.000) or strategy in SWM (MDD vs. HC: *p* = 1.000; BD vs. HC: *p* = .271; MDD vs. BD: *p* = .068) for post hoc group comparisons (Table 2).

**Table 2:** The result of ANCOVAs for the raw score of neurocognitive assessment between the three groups

		MDD (*n* = 30)	BD (*n* = 30)	HC (*n* = 30)	*F*	*p*	Post hoc	Effects of covariates (*t*, *p*)			
								Age	Gender	Duration of illness	Number of depressed episodes
RTI	Five‐choice reaction time (ms)	316.5 ± 48.7	318.7 ± 51.6	284.8 ± 35.5	8.549	.000	HC < MDD, BD	4.207, .000	−0.757, .451	−3.231, .002	−0.327, .745
	Five‐choice movement time (ms)	280.5 ± 73.9	262.8 ± 57.7	244.4 ± 80.0	3.809	.026	n.s.	3.114, .003	−0.539, .591	−2.434, .017	−0.743, .460
RVP	A′	0.92 ± 0.06	0.88 ± 0.05	0.93 ± 0.05	4.007	.022	MDD, HC > BD	−3.542, .001	−2.343, .021	−0.384, .702	0.623, .535
	Median response latency (ms)	542.6 ± 125.0	536.8 ± 140.2	481.9 ± 85.6	1.315	.274		5.232, .000	1.270, .208	−0.202, .841	0.762, .449
IED	Total errors adjusted	15.3 ± 14.1	35.3 ± 37.9	12.8 ± 11.0	5.429	.006	MDD, HC < BD	3.387, .001	1.333, .186	−1.227, .223	0.589, .558
	Stages completed	8.2 ± 1.0	8.0 ± 1.4	8.2 ± 1.0	1.519	.225		−3.658, .000	−1.540, .127	0.193, .160	0.193, .847
SWM	Between‐errors	35.7 ± 23.2	42.4 ± 22.4	30.0 ± 20.0	0.125	.882		3.796, .000	3.506, .001	−0.062, .950	3.231, .002
	Strategy	33.1 ± 6.6	36.5 ± 5.2	34.0 ± 5.3	3.402	.038	n.s.	4.776, .000	3.468, .001	1.633, .106	3.144, .002

Abbreviations: ANCOVA, analysis of covariance; BD, bipolar disorder; HC, healthy control; IED, intra/extradimensional shift task; MDD, major depressive disorder; ms, milliseconds; *n*, number; n.s., not significant; RTI, reaction time; RVP, rapid visual processing; *SD*, standard deviation; SWM, spatial working memory; yr, year.

### 3.3 Comparison of mean FA between groups with DTI whole‐brain tractography

**Figure 1:**
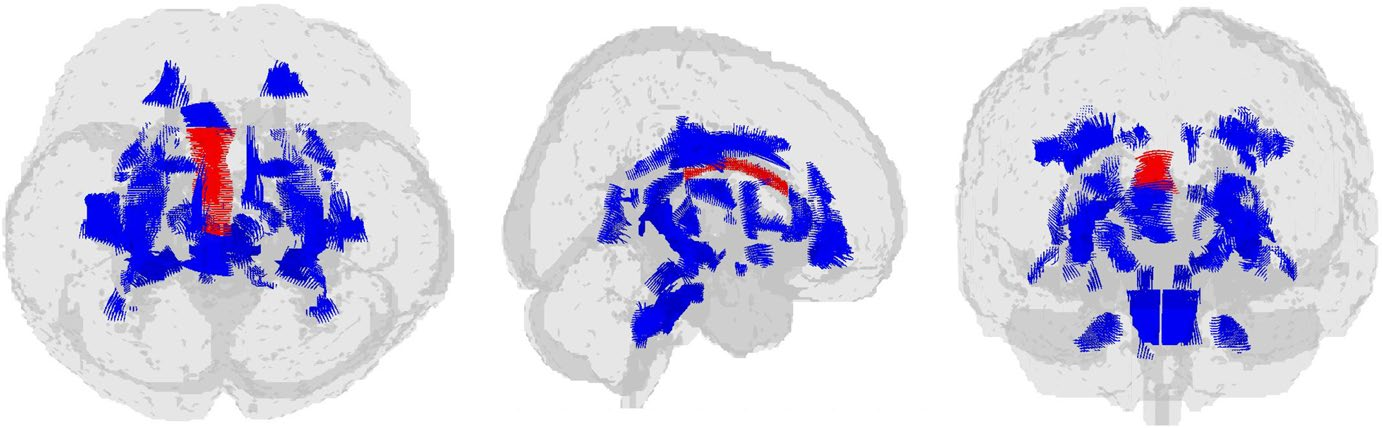
Glass brain view of the difference in FA signal depicted in red among BD, MDD, and HC in top, right, and front views. Patients with BD show significantly lower mean FA values than the patients with MDD as well as the HCs in the bilateral body of corpus callosum. BD, bipolar disorder; FA, fractional anisotropy; HC, healthy controls; MDD, major depressive disorder

Significant ANCOVA group effects were detected for FA in the bilateral body of the corpus callosum after correcting for multiple comparisons (*p* < .001). At the post hoc analysis, the participants with BD had significantly lower mean FA values than the participants with MDD as well as the HCs (left: MDD > BD: *p* = .002; HC > BD: *p* = .001, right: MDD > BD: *p* < .001; HC > BD: *p* = .001) (Figure 1, Supporting Information).

### 3.4 Correlations between FA values and neurocognitive functions

**Figure 2:**
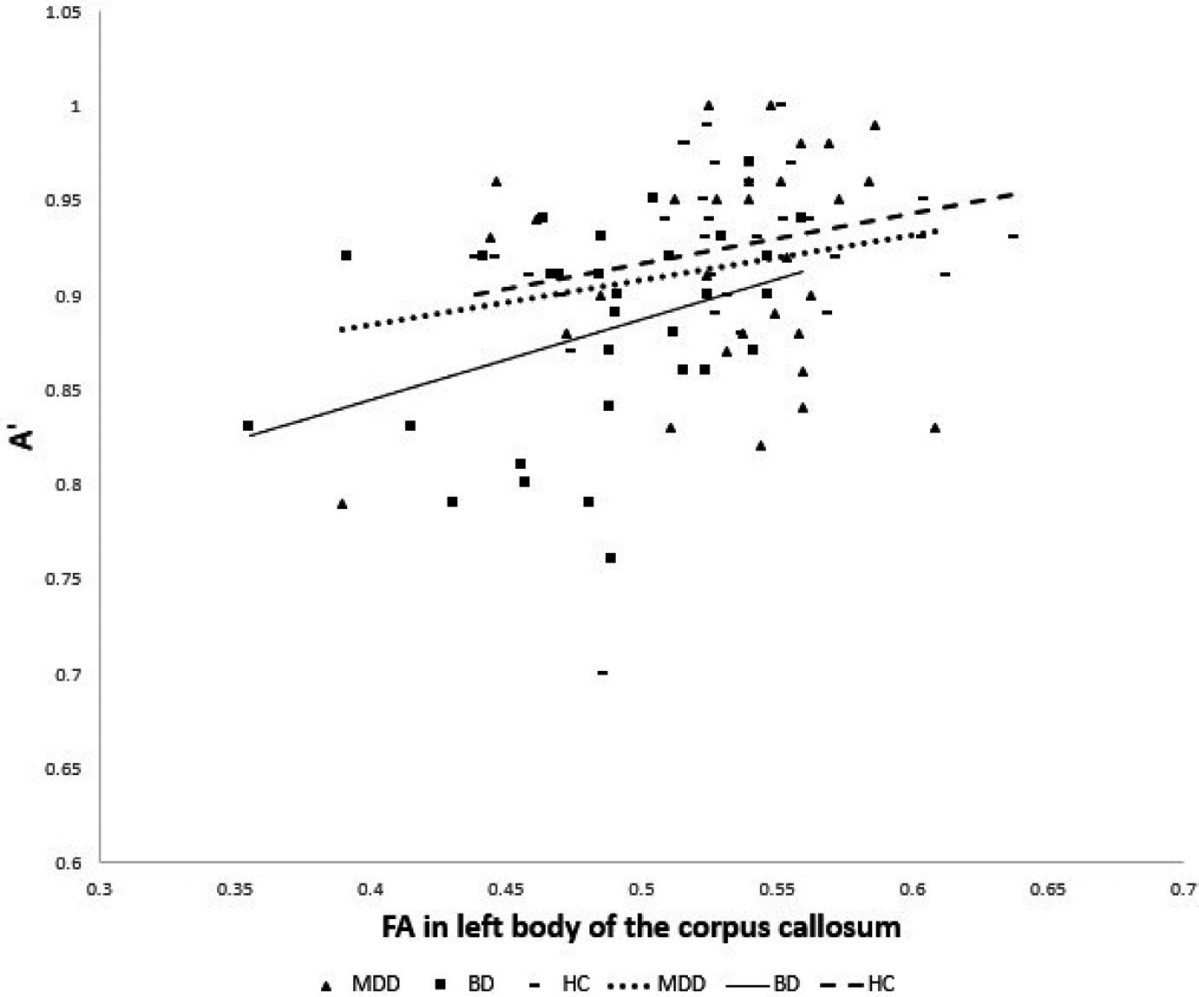
Correlation between the raw score of A′ in RVP and the FA value in the left body of the corpus callosum. The dotted, solid, and dashed lines indicate the correlations between groups. The raw score of A′ in RVP is positively correlated with the FA value in the left body of the corpus callosum in subjects with BD. BD, fractional anisotropy; FA, fractional anisotropy; RVP, Rapid visual processing

Significant positive correlations were detected between the mean FA value of the bilateral body of the corpus callosum and the raw A′ scores for all subjects. We also obtained a significant positive correlation between the mean FA value for the left body of the corpus callosum and the raw A′ score for participants with BD. There was no significant correlation between the mean FA value of the bilateral body of the corpus callosum and the raw five‐choice RTI or the total raw errors adjusted (Table 3., and Figure 2.).

**Table 3:** Correlation between the FA in body of the corpus callosum and the raw score of neurocognitive assessment for every three groups and all subjects

	RTI: five‐choice reaction time				RVP: A′				IED: Total errors adjusted			
	MDD	BD	HC	Total	MDD	BD	HC	Total	MDD	BD	HC	Total
Body of corpus callosum L	*r* = −0.344	*r* = −0.161	*r* = 0.010	*r* = −0.182	*r* = 0.196	*r* = 0.387*	*r* = 0.237	*r* = 0.363**	*r* = −0.118	*r* = −0.096	*r* = 0.104	*r* = −0.184
Body of corpus callosum R	*r* = −0.336	*r* = 0.042	*r* = −0.052	*r* = −0.161	*r* = 0.021	*r* = 0.293	*r* = 0.098	*r* = 0.260*	*r* = 0.004	*r* = −0.121	*r* = 0.214	*r* = −0.195

Abbreviations: BD, bipolar disorder; FA, fractional anisotropy; HC, healthy control; IED, intra/extradimensional shift task; L, left; MDD, major depressive disorder; *r*, correlation coefficient;R, right; RTI, reaction time; RVP, rapid visual processing.

* Significant at *p* < .05.

** Significant at *p* < .001.

## 4 DISCUSSION

The purpose of this study was to comprehensively assess white matter tracts in patients with MDD and BD during euthymia using whole‐brain tractography and to examine the pathophysiological significance of different degrees of white matter abnormalities between the two disorders from the perspective of cognitive function.

Results indicated that patients with BD had a significant reduction of FA in the bilateral body of the corpus callosum compared to patients with MDD and HCs, even after multiple comparison correction, and controlling for age, gender, duration of illness, and number of episodes as covariates. Similarly, patients with BD showed a significant dysfunction in sustained attention and set‐shifting. FA in the bilateral body of the corpus callosum was correlated with sustained attention scores in patients with BD and the total sample of participants. On the other hand, there was no correlation between FA and set‐shifting scores for any of the groups. It is suggested that a more significant abnormality in the body of the corpus callosum of patients with BD might be related to the dysfunction in sustained attention.

Different to the results of the current study, Yamada et al. (2015) reported white matter abnormalities in the anterior body of the corpus callosum of patients with MDD and BD, as well as cognitive dysfunction in working memory, attention, and verbal memory in both groups, suggestive of no differences in white matter integrity, or cognitive functioning between the two disorders. Their study included patients with euthymia and depression, whereas only euthymic patients were assessed in the current study. It has been reported that white matter integrity fluctuates according to the mood state (Magioncalda et al., 2016; Taylor et al., 2011; Zanetti et al., 2009), although these abnormalities remain during euthymic periods (Arnold et al., 2012; Chaddock et al., 2009; Yurgelun‐todd et al., 2007) Likewise, some aspects of cognitive dysfunction in mood disorders persist during euthymia (Clark et al., 2002; Holmes et al., 2008), and some recover (Clark et al., 2005; Liu et al., 2002; Maalouf et al., 2010). Therefore, it is possible that Yamada et al.’s results were affected by the data of patients in a depressive state, whereas the results of this study more directly reflected the condition of euthymic patients. Indeed, our results agree with studies that directly compared euthymic patients with MDD and BD, and reported greater abnormalities in the corpus callosum white matter integrity (Benedetti et al., 2011; Repple et al., 2017), and sustained attention (Clark et al., 2005; Maalouf et al., 2010) of BD patients. Moreover, we comprehensively examined 54 white matter tracts in the whole brain, but we could not find any significant reductions in the white matter except in the corpus callosum. Therefore, the corpus callosum appears to play an important role in the pathology of BD.

Sustained attention is defined as the ability to maintain attention for prolonged periods of time and to react efficiently to unpredictable stimuli. Deficits of sustained attention in psychiatric patients might adversely affect their psychological and occupational functioning, and correlate with a worse prognosis and a poor quality of life (Harmer et al., 2002; Sarter et al., 2001). Recent task‐based MRI and functional MRI studies have indicated that sustained attention is controlled by multiple neural structures that together represent a brain network. There is widespread agreement that the dorsal attention network (DAN) is a representative brain network that supports sustained attention. The DAN includes the following four regions: intraparietal sulcus (IPS)/superior parietal lobule (SPL); superior precentral sulcus (sPCS) containing the putative human homologues of the nonhuman primate frontal eye field (FEF); inferior precentral sulcus (iPCS), also referred to as inferior frontal junction; and the motion‐sensitive area MT complex (Brissenden et al., 2016). The corpus callosum is the largest white matter structure connecting the cerebral hemispheres, which provides interhemispheric integration and the transfer of information (Fabri & Polonara, 2013). According to the description by Witelson (1989), the body of the corpus callosum can be geometrically subdivided into four regions: the rostral body, anterior midbody, posterior midbody, and isthmus. The topographical organization of fibers coursing through the corpus callosum has been described by electrophysiological and neuroanatomical studies of nonhuman primates, human lesions studies, and functional MRI mapping studies (Fabri & Polonara, 2013). The anterior part of the corpus callosum body, including the rostral body and anterior midbody, interconnects the frontal and premotor cortices, the posterior midbody interconnects the somatosensory cortex, and the isthmus interconnects the parietal and superior temporal cortices (Fabri, 2014; Sakai et al., 2017). Although there are few previous studies that have indicated the relationship between DAN and the corpus callosum, it has been suggested that the corpus callosum plays an important role in attentional functions (Banich, 1998; Hines et al., 2002; Huang et al., 2015). Moreover, the corpus callosum plays a role in the interhemispheric transfer of information between the various DAN regions, such as the sPCS and the iPCS located in the frontal cortex and the IPS/SPL located in the parietal cortex. Further studies are required to examine the relationship between DAN and the corpus callosum.

Several limitations of this study must be noted. First, patients with BD have longer illness durations and a greater number of illness episodes than patients with MDD. We included both these variables in our statistical analysis as covariates; however, correlations have been reported between the illness duration and FA in patients with MDD (De Diego‐Adeliño et al., 2014), and therefore, we need to consider the possible influence of this correlation on white matter integrity. Second, all the patients were on medication when they were scanned. Specific studies have reported associations between psychotropic drugs and white matter integrity (Bracht et al., 2015b; Versace et al., 2008), whereas others have reported no such associations (Bracht et al., 2014; Cullen et al., 2010), which makes the results on the association between psychotropic drugs and white matter integrity controversial. It has also been reported that mood stabilizers, which are a core aspect in the treatment of BD, affect myelination and its plasticity and repair as a glycogen synthase kinase 3β inhibitor (Bartzokis, 2012; Makoukji et al., 2012). Controlling for demographic variables and the recruitment of a medication‐free patient sample are required to verify the results of this study. Finally, multiple comparisons were made with the use of the Bonferroni method, which tends to be a conservative test, and therefore, the possibility of underestimation needs to be considered.

## 5 CONCLUSION

This study is the first to compare white matter integrity between euthymic patients with MDD and BD and examine the pathophysiological significance of the difference in white matter integrity from the perspective of cognitive function. We comprehensively explored the whole brain, and as a result, we found a significant reduction in white matter integrity only in the corpus callosum of patients with BD, and this abnormality was associated with an impairment of sustained attention. We believe that these results would contribute to the improved understanding of the neurostructural basis of cognitive dysfunction in mood disorders, as well as to the development of intervention methods for this condition.

## DISCLOSURES

This research was supported by AMED under Grant Numbers JP18dm0207012 and JP18dm0107093. This research was also partially supported by “Research and development of technology for enhancing functional recovery of elderly and disabled people based on non‐invasive brain imaging and robotic assistive devices” of the Commissioned Research of National Institute of Information and Communications Technology (NICT, Grant Number 1870110) and AMED under Grant Number JP18dm0307002.

## CONFLICT OF INTEREST

None of the authors have any conflicts of interest to declare regarding the findings of this study.

## AUTHOR CONTRIBUTION

Masuda designed the study and wrote the initial draft of the manuscript. Okuhata and Kobayashi contributed to processing and analysis of MRI data, and assisted in the preparation of the manuscript. All other authors have contributed to data collection and interpretation, and critically reviewed the manuscript. All authors approved the final version of the manuscript and agreed to be accountable for all aspects of the work in ensuring that questions related to the accuracy or integrity of any part of the work are appropriately investigated and resolved.

### Peer Review

The peer review history for this article is available at https://publons.com/publon/10.1002/brb3.1868.

## Supporting information

Supplementary Material

## Data Availability

Due to potentially identifying information, the data for this study are ethically restricted by the Ethical Committee for Epidemiology of Hiroshima University, Japan. Interested, qualified researchers may request the data by contacting Dr. Shoji Karatsu (kasumi‐kenkyu@office.hiroshimau.ac.jp).
