# Supplementary material for: White matter abnormalities and cognitive function in euthymic patients with bipolar disorder and major depressive disorder
Source: Brain Behav. 2020 Oct 3;10(12):e01868. doi: 10.1002/brb3.1868 (PMC7749556; doi:10.1002/brb3.1868)
Supplement: Supplementary file 1 — Supplementary Material [file BRB3-10-e01868-s001.docx]

| Supplement 1. The result of ANCOVAs for FA in 54 white matter parcellations between the three groups. | | | | | | | | | | | |
| --- | --- | --- | --- | --- | --- | --- | --- | --- | --- | --- | --- |
|  | FA | | |  | | | effects of covariates (t, P) | | | |  |
|  | MDD | BD | HC | F | P | Post hoc | age | gender | duration of  illness | number of  depressed episodes |  |
| Corticospinal tract L | 0.499±0.029 | 0.493±0.033 | 0.500±0.035 | 0.328 | 0.721 |  | 2.226, 0.029 | -3.167, 0.002 | -0.732, 0.466 | -1.592, 0.115 |  |
| Corticospinal tract R | 0.510±0.038 | 0.518±0.031 | 0.533±0.035 | 3.491 | 0.035 |  | 0.921, 0.360 | -3.617, 0.001 | 0.149, 0.882 | -1.025, 0.308 |  |
| Inferior cerebellar peduncle L | 0.379±0.031 | 0.377±0.035 | 0.386±0.028 | 0.642 | 0.529 |  | 1.136, 0.259 | 0.712, 0.479 | -0.202, 0.84 | -0.712, 0.478 |  |
| Inferior cerebellar peduncle R | 0.431±0.030 | 0.428±0.030 | 0.428±0.026 | 0.064 | 0.938 |  | 1.909, 0.060 | -0.934, 0.353 | 0.512, 0.61 | -0.844, 0.401 |  |
| Medial lemniscus L | 0.518±0.025 | 0.509±0.023 | 0.521±0.025 | 2.076 | 0.132 |  | 0.990, 0.325 | -1.133, 0.260 | 0.202, 0.841 | -1.816, 0.073 |  |
| Medial lemniscus R | 0.498±0.021 | 0.492±0.028 | 0.507±0.031 | 2.637 | 0.077 |  | 0.420, 0.676 | -1.572, 0.120 | 0.061, 0.951 | -1.824, 0.072 |  |
| Superior cerebellar peduncle L | 0.494±0.024 | 0.485±0.023 | 0.493±0.020 | 1.614 | 0.205 |  | 2.635, 0.010 | -0.735, 0.465 | -1.002, 0.319 | -1.687, 0.095 |  |
| Superior cerebellar peduncle R | 0.548±0.025 | 0.542±0.024 | 0.547±0.022 | 0.496 | 0.611 |  | 3.632, 0.000 | -1.683, 0.096 | -1.216, 0.227 | -2.166, 0.033 |  |
| Cerebral peduncle L | 0.523±0.022 | 0.516±0.033 | 0.523±0.018 | 0.788 | 0.458 |  | -2.414, 0.018 | 0.578, 0.565 | -1.176, 0.243 | 0.601, 0.549 |  |
| Cerebral peduncle R | 0.555±0.028 | 0.555±0.030 | 0.557±0.024 | 0.014 | 0.986 |  | -2.571, 0.012 | -1.189, 0.238 | -0.959, 0.34 | -1.537, 0.128 |  |
| Anterior limb of internal capsule L | 0.529±0.028 | 0.524±0.036 | 0.536±0.036 | 0.967 | 0.384 |  | -3.036, 0.003 | -2.554, 0.012 | -1.117, 0.267 | 0.697, 0.488 |  |
| Anterior limb of internal capsule R | 0.540±0.022 | 0.534±0.029 | 0.542±0.031 | 0.768 | 0.467 |  | -1.549, 0.125 | -1.726, 0.088 | -1.009, 0.316 | 0.911, 0.365 |  |
| Posterior limb of internal capsule L | 0.563±0.025 | 0.562±0.024 | 0.567±0.022 | 0.300 | 0.742 |  | -0.547, 0.586 | -1.265, 0.209 | -0.422, 0.674 | -0.363, 0.718 |  |
| Posterior limb of internal capsule R | 0.600±0.029 | 0.600±0.028 | 0.600±0.028 | 0.027 | 0.973 |  | 1.141, 0.257 | -1.861, 0.066 | -0.792, 0.431 | -0.567, 0.572 |  |
| Posterior thalamic radiation L | 0.435±0.025 | 0.428±0.030 | 0.445±0.033 | 2.679 | 0.074 |  | -3.595, 0.001 | 1.101, 0.274 | -0.891, 0.376 | -0.467, 0.642 |  |
| Posterior thalamic radiation R | 0.461±0.035 | 0.437±0.041 | 0.458±0.045 | 3.160 | 0.047 |  | -4.149, 0.000 | 1.315, 0.192 | -0.408, 0.685 | -0.597, 0.552 |  |
| Anterior corona radiata L | 0.372±0.024 | 0.364±0.025 | 0.377±0.032 | 1.661 | 0.196 |  | -5.144, 0.000 | 0.363, 0.718 | -0.547, 0.586 | 1.330, 0.187 |  |
| Anterior corona radiata R | 0.367±0.026 | 0.359±0.026 | 0.371±0.027 | 1.564 | 0.215 |  | -4.785, 0.000 | 0.858, 0.394 | -0.774, 0.441 | 0.265, 0.792 |  |
| Superior corona radiata L | 0.409±0.025 | 0.403±0.027 | 0.415±0.023 | 1.853 | 0.163 |  | -1.398, 0.166 | -1.231, 0.222 | 0.089, 0.929 | -0.646, 0.520 |  |
| Superior corona radiata R | 0.434±0.020 | 0.417±0.026 | 0.434±0.021 | 5.258 | 0.007 |  | -1.295, 0.199 | -1.363, 0.177 | -0.638, 0.525 | -1.116, 0.268 |  |
| Posterior corona radiata L | 0.431±0.027 | 0.425±0.030 | 0.430±0.033 | 0.487 | 0.616 |  | -0.178, 0.859 | -0.117, 0.907 | -0.147, 0.883 | -1.020, 0.311 |  |
| Posterior corona radiata R | 0.412±0.027 | 0.398±0.037 | 0.407±0.031 | 1.530 | 0.222 |  | -2.123, 0.037 | 0.426, 0.671 | 1.096, 0.276 | 0.231, 0.818 |  |
| Cingulum (cingulate gyrus) L | 0.428±0.028 | 0.402±0.029 | 0.427±0.029 | 8.134 | 0.001 |  | -0.733, 0.466 | -2.802, 0.006 | -1.065, 0.290 | -0.414, 0.680 |  |
| Cingulum (cingulate gyrus) R | 0.440±0.034 | 0.412±0.030 | 0.441±0.033 | 7.833 | 0.001 |  | -2.414, 0.018 | -2.878, 0.005 | -1.390, 0.168 | -0.558, 0.578 |  |
| Cingulum (hippocampus) L | 0.343±0.022 | 0.327±0.034 | 0.341±0.034 | 2.473 | 0.090 |  | -1.381, 0.171 | -2.560, 0.012 | -0.662, 0.510 | -0.321, 0.749 |  |
| Cingulum (hippocampus) R | 0.368±0.035 | 0.350±0.026 | 0.358±0.040 | 2.058 | 0.134 |  | -2.848, 0.006 | -0.446, 0.657 | -0.233, 0.817 | -1.158, 0.250 |  |
| Fornix (cres) L | 0.400±0.026 | 0.393±0.029 | 0.406±0.034 | 1.630 | 0.202 |  | -4.323, 0.000 | 1.260, 0.211 | -0.342, 0.734 | 0.439, 0.662 |  |
| Fornix (cres) R | 0.441±0.034 | 0.422±0.032 | 0.436±0.040 | 2.180 | 0.119 |  | -3.574, 0.001 | 2.384, 0.019 | 0.912, 0.364 | 1.300, 0.197 |  |
| Superior longitudinal fasciculus L | 0.418±0.024 | 0.405±0.026 | 0.422±0.028 | 3.676 | 0.029 |  | 0.530, 0.598 | -2.349, 0.021 | -1.215, 0.228 | -0.887, 0.378 |  |
| Superior longitudinal fasciculus R | 0.422±0.022 | 0.404±0.033 | 0.418±0.025 | 3.589 | 0.032 |  | 1.784, 0.078 | -2.560, 0.012 | -0.841, 0.403 | -0.743, 0.460 |  |
| Superior fronto-occipital fasciculus L | 0.346±0.027 | 0.342±0.039 | 0.349±0.037 | 0.324 | 0.724 |  | -3.968, 0.000 | -1.969, 0.052 | -0.845, 0.401 | 0.258, 0.797 |  |
| Superior fronto-occipital fasciculus R | 0.346±0.030 | 0.346±0.035 | 0.349±0.043 | 0.092 | 0.912 |  | -4.127, 0.000 | -1.288, 0.201 | -0.713, 0.478 | 1.109, 0.271 |  |
| Inferior fronto-occipital fasciculus L | 0.399±0.024 | 0.394±0.030 | 0.413±0.032 | 3.717 | 0.028 |  | -1.689, 0.096 | -1.223, 0.225 | -1.283 0.203 | 0.106, 0.916 |  |
| Inferior fronto-occipital fasciculus R | 0.383±0.023 | 0.380±0.026 | 0.397±0.031 | 3.158 | 0.047 |  | -2.056, 0.043 | 0.081, 0.936 | -0.856, 0.394 | -1.002, 0.319 |  |
| Sagittal stratum L | 0.423±0.024 | 0.420±0.032 | 0.424±0.023 | 0.17 | 0.844 |  | -1.925, 0.058 | -0.497, 0.62 | -0.464, 0.644 | 1.112, 0.270 |  |
| Sagittal stratum R | 0.435±0.030 | 0.435±0.024 | 0.432±0.033 | 0.093 | 0.911 |  | -3.363, 0.001 | 1.760, 0.082 | 0.745, 0.458 | 0.306, 0.760 |  |
| External capsule L | 0.280±0.021 | 0.2751±0.032 | 0.284±0.020 | 0.974 | 0.382 |  | -2.572, 0.012 | -1.105, 0.272 | -2.280, 0.025 | -0.235, 0.815 |  |
| External capsule R | 0.329±0.027 | 0.328±0.026 | 0.336±0.025 | 0.860 | 0.427 |  | -3.664, 0.000 | 1.782, 0.078 | -1.589, 0.116 | 0.826, 0.411 |  |
| Uncinate fasciculus L | 0.317±0.031 | 0.317±0.032 | 0.329±0.027 | 2.431 | 0.094 |  | -0.042, 0.967 | -1.479, 0.143 | 0.710, 0.480 | 0.593, 0.554 |  |
| Uncinate fasciculus R | 0.250±0.039 | 0.253±0.035 | 0.265±0.039 | 1.483 | 0.233 |  | 0.534, 0.595 | 0.093, 0.926 | -0.632, 0.529 | -0.220, 0.826 |  |
| Pontine crossing tract L | 0.458±0.032 | 0.467±0.035 | 0.482±0.029 | 4.437 | 0.015 |  | 0.067, 0.947 | -0.312, 0.755 | 2.353, 0.021 | -0.398, 0.692 |  |
| Pontine crossing tract R | 0.421±0.027 | 0.414±0.038 | 0.394±0.027 | 6.058 | 0.003 |  | -1.161, 0.249 | 0.109, 0.914 | 2.134, 0.036 | -0.579, 0.564 |  |
| Middle cerebellar peduncle L | 0.473±0.013 | 0.468±0.020 | 0.475±0.023 | 1.082 | 0.343 |  | -0.773, 0.442 | -2.533, 0.013 | 0.762, 0.448 | -0.909, 0.366 |  |
| Middle cerebellar peduncle R | 0.477±0.019 | 0.478±0.020 | 0.483±0.022 | 0.743 | 0.478 |  | -2.844, 0.006 | -4.784, 0.000 | 1.473, 0.145 | -1.874, 0.064 |  |
| Fornix (column and body of fornix) L | 0.217±0.060 | 0.165±0.053 | 0.194±0.077 | 4.917 | 0.009 |  | -6.833, 0.000 | 2.831, 0.006 | 1.300, 0.197 | 1.927, 0.057 |  |
| Fornix (column and body of fornix) R | 0.312±0.084 | 0.230±0.090 | 0.264±0.096 | 6.222 | 0.003 |  | -4.931, 0.000 | 1.861, 0.066 | 0.080, 0.937 | 0.423, 0.674 |  |
| Genu of corpus callosum L | 0.588±0.039 | 0.564±0.046 | 0.591±0.040 | 3.541 | 0.033 |  | -4.913, 0.000 | 0.806, 0.423 | -0.293, 0.770 | -0.277, 0.783 |  |
| Genu of corpus callosum R | 0.605±0.044 | 0.576±0.050 | 0.606±0.044 | 4.054 | 0.021 |  | -4.502, 0.000 | 1.130, 0.262 | -0.168, 0.867 | -0.183, 0.855 |  |
| Body of corpus callosum L | 0.531±0.048 | 0.487±0.047 | 0.532±0.047 | 8.681 | **0.000** | MDD,HC > BD | -4.522, 0.000 | 1.759, 0.082 | -1.086, 0.280 | 0.614, 0.541 |  |
| Body of corpus callosum R | 0.516±0.050 | 0.465±0.051 | 0.514±0.048 | 10.045 | **0.000** | MDD,HC > BD | -3.152, 0.002 | 2.146, 0.035 | -1.088, 0.280 | 0.273, 0.786 |  |
| Splenium of corpus callosum L | 0.624±0.023 | 0.613±0.026 | 0.630±0.025 | 3.539 | 0.033 |  | -1.467, 0.146 | 0.717, 0.476 | 0.177, 0.860 | 0.208, 0.836 |  |
| Splenium of corpus callosum R | 0.670±0.032 | 0.653±0.033 | 0.672±0.034 | 2.953 | 0.057 |  | -3.031, 0.003 | 1.386, 0.170 | 0.519, 0.605 | 0.495, 0.622 |  |
| Retrolenticular part of internal capsule L | 0.436±0.019 | 0.430±0.018 | 0.432±0.024 | 0.744 | 0.478 |  | -2.371, 0.020 | -0.116, 0.908 | 0.169, 0.866 | 0.413, 0.681 |  |
| Retrolenticular part of internal capsule R | 0.444±0.025 | 0.435±0.020 | 0.440±0.025 | 1.279 | 0.283 |  | -1.431, 0.156 | -0.386, 0.701 | -0.544, 0.588 | -0.615, 0.540 |  |
| Significant P-values are set in bold letters after multiple comparison correction (p<0.0093). Abbreviation: FA = fractional anisotropy, MDD = major depressive disorder, BD = bipolar disorder, HC = healthy control, SD = standard deviation, R = right, L = left. | | | | | | | | | | | |
